# Homozygous Duplication in the *CHRNE* in a Family with Congenital Myasthenic Syndrome 4C: 18-Year Follow Up

**DOI:** 10.3390/biomedicines11112983

**Published:** 2023-11-06

**Authors:** Ahmad M. Almatrafi, Majed M. Alluqmani, Sulman Basit

**Affiliations:** 1 Department of Biology, College of Science, Taibah University, Medina 42353, Saudi Arabia; a.m.almatrafi@gmail.com; 2 Department of Neurology, College of Medicine, Taibah University, Medina 42353, Saudi Arabia; mloqmani@hotmail.com; 3 Department of Biochemistry and Molecular Medicine, College of Medicine, Taibah University, Medina 42353, Saudi Arabia

**Keywords:** congenital myasthenic syndrome, genetics, *CHRNE* mutation, phenotypic spectrum

## Abstract

Background and objectives: Congenital myasthenic syndromes (CMSs) are rare inherited diseases characterized by muscle weakness and fatigability on exertion resulting from defects in the neuromuscular junctions. Mutations in 32 genes have been reported as the underlying causes of CMS, with mutations in the cholinergic receptor nicotinic epsilon subunit (*CHRNE*) being the most common cause of the disease. Methodology and Materials: This study investigated a large consanguineous family with multiple individuals suffering from abnormal fatigue and muscle weakness in the ocular and limb regions. Moreover, the affected individuals were followed up for 18 years to observe the clinical course of the disease. Results: High-quality exome sequencing followed by bidirectional Sanger sequencing revealed a homozygous duplication variant (NM_000080.4: c.1220-8_1227dup) in the splice acceptor site of exon 11 of the *CHRNE* gene. This variant is predicted to cause frameshift and premature termination (p.Cys410ProfsTer51). Both parents had heterozygous duplication variants with no clinical symptoms. The personalized treatment of the affected individuals resulted in a marked improvement in the clinical symptoms. More than 80% of the disease symptoms in the affected individuals subsided after the use of pyridostigmine and salbutamol (4 mg). Conclusions: This is the first report of long-term follow up of cases with homozygous insertion (c.1220-8_1227dup) in the *CHRNE* gene. Furthermore, this report expands the phenotypic symptoms associated with the *CHRNE* mutation.

## 1. Introduction

Congenital myasthenic syndromes (CMSs) are a genetically heterogeneous group of disorders that result from a neuromuscular transmission defect at the motor endplate [1]. CMS is often characterized by abnormal fatigue and weakness of various muscles, such as those in the bulbar, ocular, and limb regions [2]. The onset of CMS usually occurs during fetal development, at birth, or in early childhood, particularly within the first two years of life. However, the disease can develop during adulthood on rare occasions [3].

Diagnosing CMS can be challenging due to the variability in its clinical features and causative genes [4]. The severity and progression of the condition can range from mild to severe weakness or disability, permanent weakness of muscles, respiratory deficiency, and premature death [5].

In 1977, Engel et al. recognized the first case of CMS [6]. Since then, several cases have been reported, and the disorder has been classified into three subtypes based on the location of the mutant protein within the neuromuscular junction: presynaptic, synaptic, or postsynaptic [2,5].

CMS can be effectively treated, and the therapeutic approach relies on the underlying genetic defects [7]. Typically, activated motor neurons release an excessive amount of acetylcholine (ACh), which travels through the synapse and binds to the nicotinic acetylcholine receptor (AChR), triggering muscle contraction. However, this signal can be terminated by acetylcholinesterase, which degrades ACh [8,9]. Mutations in genes encoding for the proteins involved in these signaling pathways can lead to CMS [2,9,10]. To date, mutations in 32 genes have been linked with CMS. More than half of these genes are associated with the postsynaptic AChR subunits such as *CHRNA1*, *CHRNB1*, *CHRND*, and *CHRNE* [5,7,8,9]. The *CHRNE* gene encodes the ε-subunit of the AChR. The first pathogenic variant in the *CHRNE* gene underlying CMS was identified in 2000 [11]. It is estimated that *CHRNE* mutations are responsible for up to 50% of all CMS cases, and it is the most common mutated gene in CMS patients [5,10].

The clinical features and severity of the disease associated with *CHRNE* mutations can vary among affected families. While some affected individuals display only ptosis, others may exhibit more severe clinical symptoms of generalized myasthenia [5]. Richard et al. found a single truncated mutation (c.1293insG) to be the most common cause of CMS among patients from North Africa [12]. Meanwhile, a frameshift mutation c.1267delG was documented in 60% of European patients [13]. Furthermore, compound heterozygous variants (c.295C>T and c.442T>A) were identified to cause CMS in Han Chinese [7]. Also, a heterozygous substitution mutation such as c.721C>T was found to cause the slow-channel CMS with progressive proximal–distal weakness with ocular involvement in an Italian family [14]. Interestingly, a study of two unrelated Italian families with CMS revealed phenotypic variability among patients carrying the p.T159P mutation in the *CHRNE* gene [15].

The current study reports the identification of a homozygous duplication (c.1220-8_1227dup) in the *CHRNE* gene using the whole exome sequencing (WES) approach. The variant is segregating in a family in an autosomal recessive manner. To the best of our knowledge, this is the first report of *CHRNE* in the Saudi population.

## 2. Materials and Methods

### 2.1. Human Subjects and Ethical Approval

**Figure 1:**
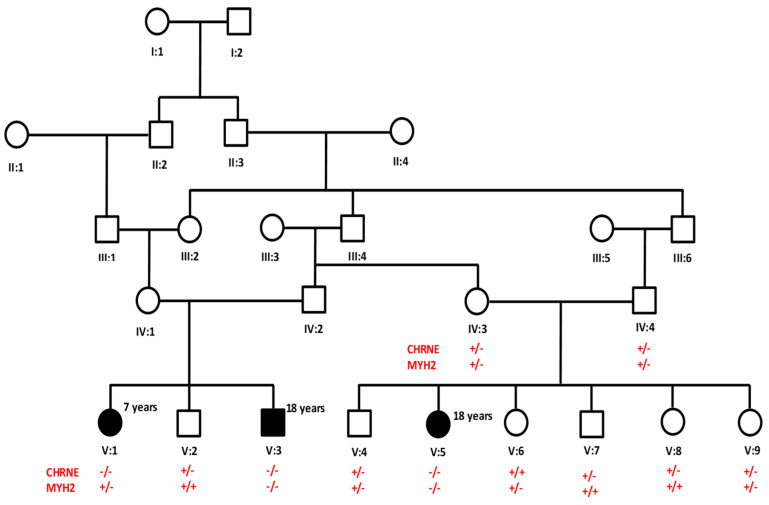
A pedigree chart of a five-generation family segregating congenital myasthenic syndromes in an autosomal recessive manner. Affected individuals are represented with black symbols and unaffected individuals with clear symbols.

A five-generation consanguineous Saudi family with one affected daughter complaining of abnormal fatigue and limb weakness was examined in neurological clinic at King Salman Medical City (Medina, Saudi Arabia). Physical examination, radiological evaluation, and laboratory investigations were carried out. Electromyography (EMG), nerve conduction studies (NCS), and magnetic resonance imaging (MRI) were carried out to diagnose the case. The family was then referred to the Center for Genetics and Inherited Diseases (CGID, Medina, Saudi Arabia) for genetic assessment. Family history was obtained through personal interviews and clinical reports. The pedigree of a family was drawn according to the information provided by the father before blood sample collection (Figure 1). The current study was approved by the Ethical Review Committee of Taibah University (TUCDREC/27032021). Written informed consent was obtained from all participants for genetic analysis and publication of findings.

### 2.2. Extraction of Genomic DNA and Quantification

3 mL of whole blood samples was collected from each participant in EDTA-containing blood vacutainers. The participants included three affected individuals (V:1, V:3, and V:5), and eight unaffected members (IV:3, IV:4, V:4, V:6, V:7, V:8, V:9, and V:2). Subsequently, genomic DNA was extracted via the QiaAmp DNA mini kit (Qiagen cat no. 51306) following the manufacturer’s instructions. The purity and integrity of each DNA sample were determined using a Qubit fluorometer and a Nanodrop-1000 spectrophotometer (Thermo Fisher Scientific, Waltham, MA, USA).

### 2.3. Exome Sequencing

Whole exome sequencing (WES) was performed using a DNA sample of an affected individual (V:5) to identify the underlying genetic variant(s). The preparation of DNA libraries was performed using the SureSelect kit, and sequence reads were generated via the Illumina Hiseq 2000/2500 machine. Reads were analyzed and filtered following the protocol described elsewhere [16,17]. Since the disorder followed an autosomal recessive inheritance pattern, only homozygous and compound heterozygous variants were filtered based on the family pedigree and consanguineous marriages. The variants were classified according to the ACMG guidelines [18].

### 2.4. Sanger Sequencing and In Silico Analysis

Sanger sequencing was employed to analyze all candidate variants identified in the patient DNA sample (V:5) across all the family members. Primer3 software (version 0.4.0) was utilized to design primers for the candidate genes. Variant pathogenicity was evaluated using Varsome (https://varsome.com/, accessed on 15 April 2023). Moreover, ClinVar was searched for this variant.

UNIPROT (https://www.uniprot.org, accessed on 15 April 2023) was utilized to map the identified pathogenic variant to the CHRNE domain across other species. In addition, the STRING website (https://version-12-0.string-db.org/, accessed on 15 April 2023) was used to investigate the CHRNE interaction network protein.

## 3. Results

### 3.1. Clinical Assessment

An 18-year-old girl (V:5) is the second child of healthy parents who are first-degree cousins. Her medical history showed that she was delivered as a full-term baby after a normal pregnancy and has never experienced any brain injury, accidents, or emotional trauma. There was no need for ICU admission after her delivery. Her parents and healthy siblings showed normal growth and development without any abnormal neurological features or muscle weakness. The initial symptoms, noticed 2–3 days after delivery, included weak abnormal sucking, low-pitched-intensity crying, dysphagia, feeding difficulties, and bronchial hypersecretion. During the first 3 months, she experienced excessive nasal discharge, dysphagia, stridor, and upper airway obstruction, the latter especially during sleep. She also had frequent choking and coughing with extreme sputum while asleep and faced breastfeeding difficulties. At the age of 3 months, she started developing moderate bilateral ptosis, with fixed eyes, and was especially unable to move her eyes laterally to the right or left side. She was examined by a neurology and genetics consultant, followed by further clinical investigations using electromyography (EMG), nerve conduction studies (NCS), and magnetic resonance imaging (MRI). EMG, NCS, and MRI examinations were within the normal range. Additionally, her metabolic and blood profiles did not show any abnormalities. When she reached 16 months of age and started walking, her father (a consultant pediatrician) noticed she had generalized hypotonia and joint laxity, especially in the knee, elbow, and wrist joints. By the age of 18 months, she had gait abnormalities and difficulty in standing, requiring support to stand. At the age of 3 years, she underwent adenectomy and tonsillectomy surgeries due to her dysphagia, dysphonia, stridor, and intensive secretions, especially during viral infections, which caused a change in her voice. At the age of 7 years, she was diagnosed with myasthenia gravis based on clinical manifestations. We performed a genetic evaluation at the age of 8 to confirm the diagnosis. A genetic diagnosis confirmed her condition as congenital myasthenic syndrome. She has shown 80–90% improvement using pyridostigmine (Mestinon 30 mg) four times per day (QID) and β2-agonists (salbutamol 2 mg) two times per day (BID) (Table 1).

Currently, she is under treatment and attends school with normal mental development. She is able to walk up to 2–3 km without any support and can climb stairs. However, in the evening, after being active throughout the day, she experiences tiredness and weakness. She still has fixed eyes and cannot move them laterally to the right or left, but her vision remains very good.

### 3.2. Genetic Analysis

An exome sequence was performed on the DNA sample of an affected individual (V:5). High-quality sequence reads were generated with more than 100× coverage. After intensive bioinformatic analysis, which includes annotation, filtration, and prioritization of the exome data, we identified a homozygous missense variant (c.5555G>T; p.Arg1852Leu) in *MYH2* gene and a likely pathogenic homozygous duplication (NM_000080.4: c.1220-8_1227dup) in the *CHRNE* gene (Table 2). This variant was deemed the most likely pathogenic variant associated with the proband clinical feature based on prior reports of *CHRNE* variants and their involvement in the development of CMS.

**Figure 2:**
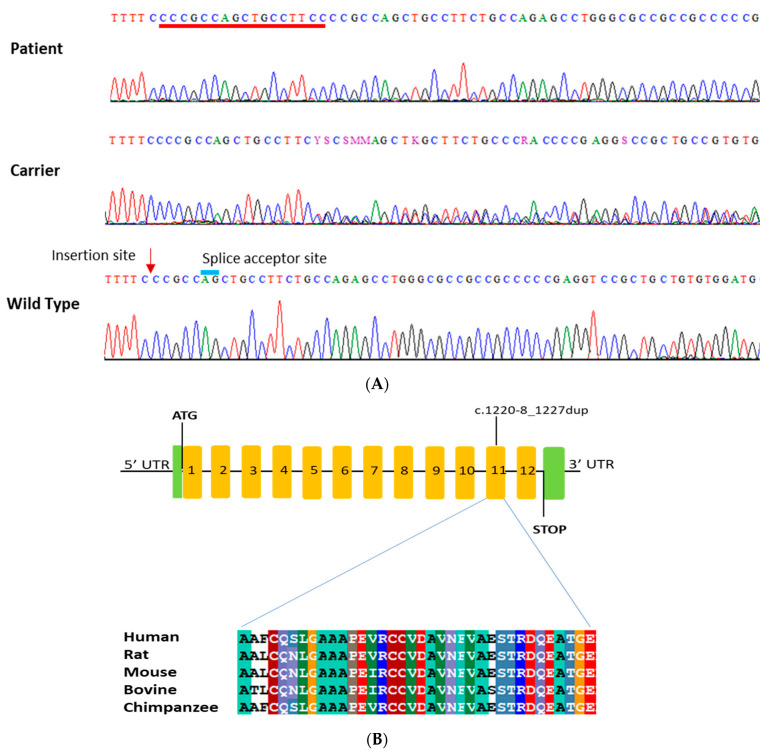
(**A**) Sanger sequence analysis of the *CHRNE* gene. Both parents (IV:3 and IV:4) and four unaffected siblings (V:4, V:7, V:8, and V:9) and one healthy cousin (V:2) carry a heterozygous variant (c.1220-8_1227dup). The affected individuals (V:5), (V:1), and (V:3) have a homozygous duplication variant underlined red. The unaffected individual (V:6) carries a wild-type sequence. Arrowhead shows the point of insertion. Blue line shows a splice acceptor site (**B**). Inserted mutation occurs in exon 11, and partial alignment of the human CHRNE amino acid in comparison with its orthologues shows a highly conserved region across species.

The 17 nucleotides’ (CCCGCCAGCTGCCTTCC) homozygous duplication (NM_000080.4: c.1220-8_1227dup) in the *CHRNE* gene is in the flanking region of the splice acceptor site of the 2nd last exon (Figure 2). This led to the insertion of extra amino acids and frameshift. This variant is predicted to be likely pathogenic by the ACMG classification (https://franklin.genoox.com/clinical-db/home, accessed on 15 April 2023) (Table 2). Also, this variant is reported in both Varsome (https://varsome.com/, accessed on 10 April 2023), and ClinVar (https://www.ncbi.nlm.nih.gov/clinvar/, accessed on 15 April 2023) as a pathogenic variant for autosomal recessive congenital myasthenic syndrome type 4A (slow-channel) in the Turkish cohort [19]. Sanger sequencing of all family members failed to show segregation of *MYH2* the variant with the phenotype. However, the *CHRNE* variant was perfectly segregating with the disease phenotype in an autosomal recessive manner. Both parents are heterozygous, and other siblings are either heterozygous or wild type for the variant without any clinical symptoms of CMS (Figure 2A). No additional pathogenic variants were identified in genes known to be associated with CMS.

### 3.3. CHRNE Protein Conservation and Interaction Network Analysis

**Figure 3:**
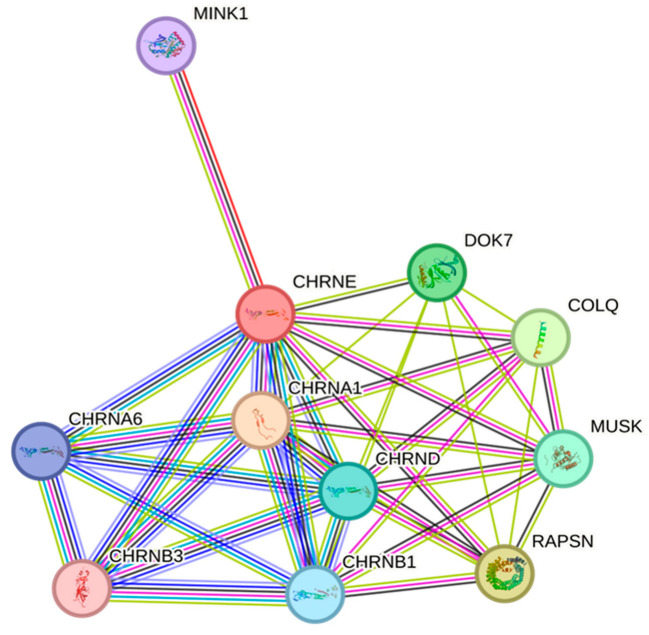
Protein–protein interaction network analysis of the CHRNE gene was conducted using the STRING database with a confidence score of 0.4. The analysis revealed a strong association between the CHRNE protein and other nicotinic acetylcholine receptor proteins, muscle-specific receptor tyrosine kinase, and neuromuscular synapses.

A highly conserved sequence of CHRNE amino acids at exon 11 was found among different species throughout evolution. Multiple amino acids’ alignments data revealed the importance of this location for the structure and function of the CHRNE protein (Figure 2B). The protein–protein interaction (PPI) study showed that CHRNE interacts with several proteins involved in skeletal muscle tissue growth and regulation of synaptic assembly at neuromuscular junctions. As depicted in Figure 3, our data revealed a network containing 11 nodes and 38 edges with a significant PPI enrichment *p*-value of 0.0000000000149. There is a strong association between the CHRNE protein and other AChR subunit proteins such as CHRNA1, CHRNA6, CHRNB1, CHRNB3, and CHRND. The interaction also involves DOK7 and MUSK proteins, which play an essential role in neuromuscular synaptogenesis.

## 4. Discussion

The majority of CMS cases share fatigable muscle weakness as a common clinical phenotype that can appear at any stage of life, from birth until adulthood [5]. However, the initial symptoms, distributions of weakness, and therapeutic response differ in early-onset conditions. This depends on specific gene mutations and the underlying molecular mechanism that disrupts signal transmission at the neuromuscular junction [20]. In the current research, clinical and genetic evaluations were performed for a family that exhibited CMS in an autosomal recessive pattern. EMG, NCS, and MRI results showed normal findings. Metabolic and blood profile analyses were also within the normal ranges. However, the proband displayed fatigable muscle weakness, bilateral ptosis, and fixed eyes. Due to the diverse characteristics of the diseases, we conducted whole-exome sequencing and identified a homozygous duplication (c.1220-8_1227dup) in the *CHRNE* gene. This mutation has been previously reported in a large cohort of CMS Turkish patients [15]. It is reported as a pathogenic variant in VarSome and ClinVar and likely pathogenic according to the ACMG franklin classification. Our results support the notion that this specific homozygous duplication (c.1220-8_1227dup) in the *CHRNE* gene is involved in the early onset of CMS.

The *CHRNE* gene is located on chromosome 17p13-p12 consisting of 12 exons, and encodes approximately 493 amino acid [21]. Mutations in the *CHRNE* gene can cause three types of CMS: type 4A (OMIM: 605809), CMS type 4B (OMIM: 616324), and CMS type 4C (OMIM: 608931). Based on the OMIM website, CMS type 4A can be inherited in an autosomal dominant (AD) or autosomal recessive (AR) manner, while types 4B and 4C show AR inherited only [22,23]. The phenotype in our cases resembles that of CMS4C. Defects in the *CHRNE* gene can lead to either acetylcholine receptor deficiency or abnormal channel kinetics [15]. The first mutation in the *CHRNE* gene associated with CMS was discovered in 2000 [22]. To date, there are 139 *CHRNE* mutations reported based on the human gene mutation database (http://www.hgmd.cf.ac.uk; accessed on 1 July 2023). This includes 63 missense mutations, 26 small deletions, 23 small insertions, 16 splicing mutations, 7 gross deletion mutations, 3 regulatory mutations, and one gross insertion and duplication. The patient diagnosed in this study has a homozygous duplication NM_000080.4:c.1220-8_1227dup in *CHRNE*. The 17 nucleotide CCCGCCAGCTGCCTTCC duplication at the splice acceptor site of the 2nd last exon led to the insertion of extra amino acids and frameshift (p.Cys410ProfsTer51). The last six exons of the CHRNE encode the neurotransmitter-gated ion-channel transmembrane region (https://www.kegg.jp/ssdb-bin/ssdb_motif?kid=hsa:1145, accessed on 23 May 2023), and it is a part of the four transmembrane helices that form the ion channel (https://www.genome.jp/dbget-bin/www_bget?pf:Neur_chan_memb, accessed on 23 May 2023). Frameshift mutation and premature termination (PTC) due to the insertion variant, identified in this study, is the most possible prediction based on various tools. This PTC may lead to the activation of nonsense-mediated decay (NMD) of the defective messenger RNAs (mRNA) [23,24], and thus the absence of CHRNE protein. However, if the mRNA somehow escapes NMD, the resulting protein is predicted to be without an extracellular domain. Th extracellular cap domain of channel proteins usually forms a dome above the extracellular entrance of the channel [25]. The absence of the cap, due to the frameshift variant, increases the average conductance and affects ion selectivity [24,25].

Both affected cousins (V:1 and V:3) exhibit similar clinical features to our patient, and the Sanger sequencing performed on both individuals revealed a similar insertion. The amino acid alignment result showed that the mutant region of the CHRNE is highly conserved among various species, such as rats, mice, bovines, and chimpanzees. The study of the PPI interaction network revealed that CHRNE interacts with several vital cellular proteins. For instance, CHRNE interacts with DOK7, which is essential for neuromuscular synaptogenesis [26,27]. Additionally, it interacts with the MINK1 protein, which acts as a coupling cell surface receptor [28]. Furthermore, MUSK is highly interacted with CHRNE, and this protein is a muscle-skeletal tyrosine kinase [29].

Acetylcholine-esterase inhibitor (AchEI) treatment is usually more effective in most CMS patients [30]. Nevertheless, in some cases, using pyridostigmine and 3,4-DAP may be ineffective and might cause worse symptoms [31]. Salbutamol alone or in combination with Fluoxetine shows a significant improvement phenotype [32]. Our patient is undergoing therapy using both pyridostigmine (Mestinon 30 mg PO every QID) and β2-agonists (salbutamol 2 mg PO every BID). Clinical improvement of up to 80% has been observed following treatment. She can walk up to 2–3 km, and improvements in bulbar, ptosis, and fatigue symptoms were also noted.

## 5. Conclusions

Next-generation sequencing is a powerful tool for identifying pathogenic variants in undiagnosed cases of CMS. The identification of pathogenic variants underlying CMS is crucial for a tailored therapeutic strategy, since some CMS medications can be ineffective or even harmful to patients. The effectiveness of the treatment is dependent on two factors: the type of pathogenic variant and its kinetic defects. This study expands on the phenotypic symptoms of CMS as well as mapping the distribution of rare genetic mutations of CMS worldwide in different populations. Accurate genetic diagnosis can help distinguish CMS from seronegative autoimmune myasthenia, thereby reducing the need for immunosuppressive therapy and its potential long-term side effects.

## Figures and Tables

**Table 1:** Common clinical features found in the proband having the *CHRNE* c.1220-8_1227dup mutation.

Clinical Features	Description
Early onset	Since birth
Fluctuating symptoms	Increasing viral infection and activity
Muscle fatigue and muscular weakness	Yes, improved with treatment
Breastfeeding difficulties chokes developed post-partum	Yes, improved gradually at 9 months
Ocular muscle impairment	Ptosis and sometime squint
Bulbar symptoms	Yes, improved with treatment
Scoliosis	Mild
Improvement with pyridostigmine	Up to 80% improvement
Mental development	Normal
Facial weakness	None
Little functional restriction	None
Delayed motor milestones	None
High-arched palate	None

**Table 2:** Bioinformatic analysis of the mutation identified in the CHRNE gene via whole exome sequencing.

Gene	Nucleotide Variant	Exon	Protein Variant	Zygosity	gnomAD Exome Freq	ACMG Franklin	VarSome	ClinVar
*CHRNE*	c.1220-8_1227dup	11	N/A	Homo	Not reported	Likely Pathogenic	Pathogenic	Pathogenic

## Data Availability

The data that support the findings of this study are available on request from the corresponding author.
